# A review of multivariate analyses in imaging genetics

**DOI:** 10.3389/fninf.2014.00029

**Published:** 2014-03-26

**Authors:** Jingyu Liu, Vince D. Calhoun

**Affiliations:** ^1^The Mind Research Network and Lovelace Biomedical and Environmental Research InstituteAlbuquerque, NM, USA; ^2^Department of Electrical and Computer Engineering, University of New MexicoAlbuquerque, NM, USA

**Keywords:** imaging genetics, multivariate analyses, genotype, phenotype, intermediate phenotypes

## Abstract

Recent advances in neuroimaging technology and molecular genetics provide the unique opportunity to investigate genetic influence on the variation of brain attributes. Since the year 2000, when the initial publication on brain imaging and genetics was released, imaging genetics has been a rapidly growing research approach with increasing publications every year. Several reviews have been offered to the research community focusing on various study designs. In addition to study design, analytic tools and their proper implementation are also critical to the success of a study. In this review, we survey recent publications using data from neuroimaging and genetics, focusing on methods capturing multivariate effects accommodating the large number of variables from both imaging data and genetic data. We group the analyses of genetic or genomic data into either *a priori* driven or data driven approach, including gene-set enrichment analysis, multifactor dimensionality reduction, principal component analysis, independent component analysis (ICA), and clustering. For the analyses of imaging data, ICA and extensions of ICA are the most widely used multivariate methods. Given detailed reviews of multivariate analyses of imaging data available elsewhere, we provide a brief summary here that includes a recently proposed method known as independent vector analysis. Finally, we review methods focused on bridging the imaging and genetic data by establishing multivariate and multiple genotype-phenotype-associations, including sparse partial least squares, sparse canonical correlation analysis, sparse reduced rank regression and parallel ICA. These methods are designed to extract latent variables from both genetic and imaging data, which become new genotypes and phenotypes, and the links between the new genotype-phenotype pairs are maximized using different cost functions. The relationship between these methods along with their assumptions, advantages, and limitations are discussed.

## INTRODUCTION

While most genetic studies have focused on phenotypes as diagnoses and clinical symptoms, it is relatively recent that intermediate phenotypes have become an ever increasing focus. Intermediate phenotypes refer to biological trait phenotypes conveying relatively closer association or higher penetration than traditional phenotypes (Meyer-Lindenberg and Weinberger, 2006; Rasetti and Weinberger, 2011). The best examples of approaches leveraging intermediate phenotypes come from studies of psychiatric disorders for which diagnoses are based mainly on clinical observations and interviews. Intermediate phenotypes derived from neuroimaging and signals directly assessing brain structure and function not only reduce the phenotypic heterogeneity common to many psychiatric disorders, but also increase detection power, given the genetic effects are not expressed directly as behaviors but as molecular and cellular functions mediating brain development and processes (Gottesman and Gould, 2003; Rose and Donohoe, 2013). The pioneer studies utilizing neuroimaging features to identify genetic impact were in the year 2000 (Bookheimer et al., 2000; Heinz et al., 2000; Small et al., 2000). They signified the birth of a new research approach using imaging genetics. As defined (Hariri et al., 2006; Meyer-Lindenberg et al., 2008; Silver et al., 2011; Meyer-Lindenberg, 2012), it combines genetic information and neuroimaging data in the same subjects to discover neuromechanisms linked to psychiatric disorders. The overall strength of imaging genetics and its impact on psychiatric disorder studies or broader have been stated clearly in several reviews (Meyer-Lindenberg and Weinberger, 2006; Glahn et al., 2007; Bigos and Weinberger, 2010; Meyer-Lindenberg, 2010; Rasetti and Weinberger, 2011).

The overwhelming growth of imaging genetics in recent years as summarized in recent studies (Roffman et al., 2006; Bigos and Weinberger, 2010), while providing abundant promising results, also reveals challenges embedded within study designs such as validity of candidate genes, control of non-genetic confounding factors, and selection of tasks to stimulate brain specific processes. Bigos and Weinberger (2010) have provided an excellent review with applications to demonstrate the principles in designing an imaging genetic study. Another big challenge faced by both imagers and geneticists is how to properly analyze the collected data, since both neuroimaging and genetics tend to generate a large amount of data. Different strategies, processing approaches, and validation methods such as false positive control (Silver et al., 2011) have been implemented and tailored for different conditions. But there is an even greater need in the future for the methodology development as pointed by Mayer-Lindenberg in his recent review (Meyer-Lindenberg, 2012), where complexity of epistasis, pleiotropy and genetic by environment interactions should been considered in particular in large scale genomic studies. The availability of imaging genetic analytic tools and their proper implementation are critical for both success of individual studies and the continuing growth of imaging genetics.

The earliest imaging genetic studies focused on candidate genetic variants using either a single or a few variables (Bookheimer et al., 2000; Heinz et al., 2000; Small et al., 2000; Egan et al., 2001). For example, the dopamine transporter gene (SLC6A) was analyzed with neuroimaging data from single-photon emission computed tomography (Heinz et al., 2000). Variation within the APOE gene was associated with activities in memory function affected by Alzheimer’s disease (Bookheimer et al., 2000). COMT Val allele carriers showed increased activities in the prefrontal cortex compared to Met allele carriers (Egan et al., 2001). In parallel, the intermediate phenotypes from neuroimaging techniques can also be specified within selected brain regions or particular processes. Straightforward univariate analyses are often used and well suited for these studies. Candidate gene and candidate imaging phenotype studies in the last decade have proven the validity of imaging genetic approach as recapitulated in (Meyer-Lindenberg, 2012). But with the completion of human genome sequence and multimodal imaging practices, in conjunction with increased evidence of polygenicity and pleiotropy (Purcell et al., 2009; Sivakumaran et al., 2011; Whalley et al., 2012; Smoller et al., 2013), multivariate analysis methods are becoming more and more demanding. For instance, thousands of genetic variants have been suggested to be linked with the risk for schizophrenia (Purcell et al., 2009). Methods to capture the interactive or integrated genetic effects of a set of genetic variants, methods to extract brain networks formed from individual voxels or regions, and methods to detect, possibly, multiple genotype-phenotype connections have been developed with their limitations and advantages (Hardoon et al., 2009; Liu et al., 2009b; Vounou et al., 2010; Le Floch et al., 2012). We expect to see continued development of such powerful methods to face the challenges and promises from genome-wide whole brain association studies.

In this review, we focus on analysis approaches and, more specifically, on the multivariate analysis approaches. We will first give an overview of analysis strategies. Then, we will survey the methods and organize them according to their multivariate nature on genetic data, neuroimaging data or both.

## OVERVIEW OF ANALYSIS STRATEGIES IN IMAGING GENETICS

While various strategies can be applied to design and perform imaging genetic studies, several aspects of such studies require particular caution. Firstly, when an imaging feature is selected as the intermediate or endophenotype, useful criteria should be applied or at least considered. As summarized in (Gottesman and Gould, 2003) intermediate phenotypes should show association with illness in a population, certain level of heritability, and state-independent characters. A proper preprocessing or controlling for possible confounding factor should also be in place, such as scanning effects, age or gender difference, brain size, etc. The most often used software packages to process brain imaging data, particularly for magnetic resonance imaging (MRI) images, include FSL^1^, SPM^2^, and AFNI^3^ for functional and structural voxel-wise preprocessing, and FreeSurfer^4^ for brain regional volume and cortical thickness. Secondly, genetic data either from single genetic mutation or genomic variants should be checked for family structure, population structure, and ethnicity differences. A rationale to pull samples together should be justified through, for instance, from a homogenous group, no indication of population structure, or a proper control of ethnicity difference. The most often used software package for single nucleotide polymorphism (SNP) data is plink^5^, which provides tools to do various quality control, sample relatedness tests, filtering and population stratification. The most often used software packages (freely available) for calling copy number variation (CNV) include PennCNV^6^, and BirdSuite^7^. Even though the effect of CNVs on brain imaging phenotypes is understudied now, it has been predicted to be an important extension in the future (Meyer-Lindenberg, 2012). Thirdly, methods to test the relation between genetics and imaging phenotypes heavily rely on the dimensionality of data, as explained explicitly in next paragraph. Finally, the interpretation of results depends on the study design and analysis approaches. Keep in mind that most imaging genetic studies test the association between genetic variants and imaging phenotypes, as the analytical method itself reveals later on. Any causal relation and underlying biological mechanism is only suggestive. Particular caution should be given to genome-wise association studies which result in a set of genetic variants interactively associated with imaging phenotypes. The interaction among them, linear, non-linear, dominate, recessive, two-way or n-way, etc., needs to be carefully explained and some methods test the overall effect without knowing the detailed interrelations. The verification or at least certain levels of cross evaluation for such findings as described in (Le Floch et al., 2012) plays a very crucial role.

**FIGURE 1:**
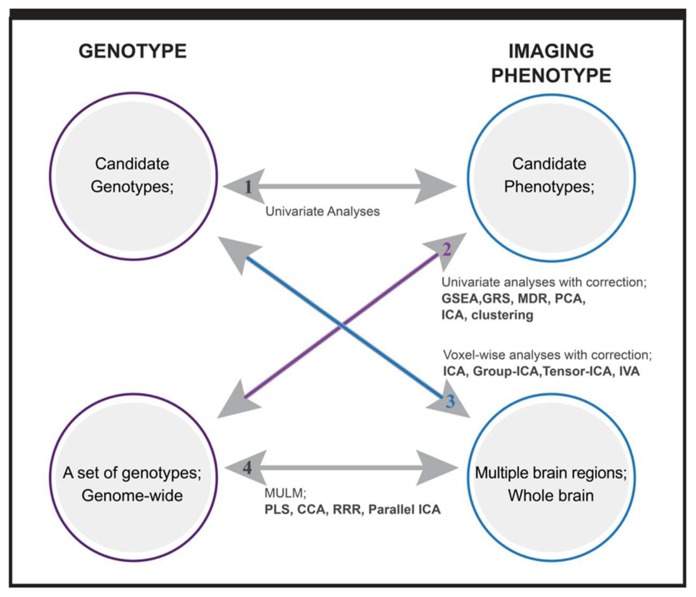
**Overview of imaging genetic studies and methods applied.** Category 1: candidate genotype with candidate phenotype. Category 2: sets of genotypes with candidate phenotype. Category 3: candidate genotype with multiple imaging phenotypes. Category 4: sets of genotypes with multiple imaging phenotypes. Methods written in bold are multivariate analysis methods. GSEA: gene set enrichment analysis; GRS: genetic risk score; MDR: multifactor dimensionality reduction; PCA: principal component analysis; ICA: independent component analysis; IVA: independent vector analysis; MULM: mass univariate linear model; PLS: partial least square; CCA: canonical component analysis; RRR: reduced rank regression.

Depending on the dimensionality of investigated genotypes and imaging intermediate phenotypes, we can classify imaging genetic studies into four categories, which is a concept borrowed from Vounou et al. (2010). As plotted in **Figure 1**, the first one includes studies with candidate phenotypes and candidate genotypes, where a direct univariate association test is applied to assess the hypothesized connection. A control for possible confounding factors (scanner, age, gender, medication, etc.) should be considered for imaging phenotypes. The second type includes studies investigating multiple genetic variants, ranging from a few to 100s of 1000s of variables in a genome-wide setting. Univariate tests corrected for multiple comparisons are straightforward (Potkin et al., 2009), but it may miss the well documented gene–gene interactions. Data driven multivariate methods and *a priori* based gene-set or pathway analyses are the two main analytical approaches to capture the interactive or integrated genetic effect (Liu et al., 2010b; Walton et al., 2013). What type of interactive relation among genes can be captured depends on the analytic methods or specifically, the models that the methods are built on. The third type includes studies investigating multiple imaging phenotypes, which may come from one or more imaging modalities, such as structural, functional MRI, magnetic resonance spectroscopy, etc. The imaging phenotypes may cover whole brain or many brain regions or voxels. Except for voxel-wise analyses with multiple comparison correction, the strategy to analyze such phenotypes usually is to extract brain networks formed by interactive brain regions or voxels, thus not only accommodating interrelations but also reducing the number of tested phenotypes (Calhoun and Adali, 2006). The last group of studies involves associations between multiple genotypic variables and multiple phenotypic variables. A typical example is genome-wide whole brain studies. Although massive univariate approaches have been implemented such as a mass-univariate linear model (MULM) in studies (Stein et al., 2010), most utilize data reduction and factorization methods to effectively capture the interactive and complex relations within and between datasets. In the following, we present the analytical methods implemented in studies of the last three categories, category 2: sets of genotypes with candidate phenotype, category 3: candidate genotype with multiple imaging phenotypes, and category 4: sets of genotypes with multiple imaging phenotypes. We focus on the multivariate approaches for each category.

## *A priori* BASED MULTIVARIATE ANALYSES ON GENETIC/GENOMIC DATA (CATEGORY 2)

Gene set enrichment analysis (GSEA) is a computational method that determines whether a prior defined set of genetic variants shows statistically significant differences between two biological states (Mootha et al., 2003; Subramanian et al., 2005) or, more generally, significant associations with phenotypes compared to the null hypothesis. The GSEA was first introduced in cancer research and thereafter various modified versions have been introduced in studies of different diseases that includes psychiatric disorders (Subramanian et al., 2005; Holden et al., 2008; Suarez-Farinas et al., 2010; Oh et al., 2011; Weng et al., 2011). The basic principle of GSEA is that sets of genetic variants are first selected for tests. We will use SNPs as an example of genetic variants without loss of generality in this review. A set of SNPs are selected based on common biological attributes (gene ontology or pathways), chromosome location, or reported results in the literature. Then the overrepresentation, or “enrichment,” of phenotype-association of this set of SNPs as one unit is calculated against the null hypothesis of normally distributed phenotype-association. Among many ways to decide the significance of enrichment (Abatangelo et al., 2009), the two most common methods are Fisher’s exact test and enrichment score test (Subramanian et al., 2005). Fisher’s exact test is fast but needs a pre-defined threshold, while enrichment score does not need a threshold but needs a permutation to get empirical *p* values. Specific issues associated, such as gene size bias (Mirina et al., 2012), linkage disequilibrium (LD) between adjacent SNPs, have been addressed by various modified versions (Liu et al., 2010b; Li et al., 2011). The rationale to select the set of SNPs comes from prior information, so this approach is indeed *a priori* driven test for the overall effect of multiple variables, without modeling the exact interaction among them. Another similar approach proposed by Walton et al. (2013) is to compute a cumulative genetic risk score $\left(GRS=\sum_{i=1}^{N}w^{i}x^{i}\right)$, which combines the additive effects of multiple SNPs selected from the continuously updated meta-analysis of genetic studies. The authors showed that this multivariate score combined the impact of many genes with small effects, accounting for 3.6% of the total variance of brain activity at dorsal lateral prefrontal cortex (Walton et al., 2013). Similar approaches using polygenic risk scores have been implemented in several other studies (Whalley et al., 2012; Smoller et al., 2013).

## DATA DRIVEN MULTIVARIATE ANALYSES ON GENETIC/GENOMIC DATA (CATEGORY 2)

Unlike the approaches above, some studies have implemented purely data driven analyses without prior information, emphasizing the genetic patterns embedded in the datasets to capture the epistasis and polygenicity. Multifactor dimensionality reduction (MDR) was developed to identify combinations of gene–gene and gene-environmental factors that are predictive of a phenotype (Hu et al., 2011; Gui et al., 2013; Pan et al., 2013). The heart of MDR is an attribute construction algorithm that creates a new variable by pooling genotypes from multiple SNPs (Moore et al., 2010). In brief, values from any combination of multiple SNPs are classified into two distinct groups, high risk and low risk, effectively reducing the dimensionality from multidimensional to one-dimensional. Subsequently, the new variables are used to identify, from all potential combinations, the specific combination of SNPs showing the strongest association with the phenotype. This method with no particular model assumption is well suited for capturing epistasis and has been used in genetics studies of various disease status (Ritchie et al., 2001; Moore and Williams, 2002; Ma et al., 2005; Lou et al., 2007; Gui et al., 2011). Extensions of the method have been developed for quantitative phenotypes and genome-wide data (Lou et al., 2007; Pattin et al., 2009; Cattaert et al., 2011; Oh et al., 2012; Winham, 2013). It is expected to see more broad applications of this method even in imaging genetics (Papassotiropoulos and de Quervain, 2011). Within the same line of estimating aggregated effect of multiple genetic variants, but based on a linear additive model, multiple regression and its penalized or modified versions have been implemented to assess the explanation power of gene variables (from a couple to genome-wide) to various of phenotypes (Wang and Abbott, 2008; Wu et al., 2009; Cule et al., 2011). Penalized regression, specifically LASSO multiple regression, are also often used to downsize variables (voxels or SNP) for further analyses (Vounou et al., 2012).

Other types of data-driven approaches, as reviewed in (Jombart et al., 2009), mainly include principal component analysis (PCA), principal coordinate analysis, non-metric dimensional scaling, and correspondence analysis, belonging to the category of matrix decomposition and extracting factors/components of weighted genetic variants. An addition to the review is independent component analysis (ICA). PCA provides a set of linearly orthogonal principal components, explaining maximal variance, while ICA is designed to extract statistically independent components (and thus uses higher order statistical information). PCA is often used in genome-wide SNP data, and the top PCs extracted most likely present the population structure helpful for population stratification (Price et al., 2006; Liu et al., 2010a). ICA has proven successful in a variety of biological inquiries when applied to gene expression data (Kong et al., 2008), including identifying tumor-related pathways (Saidi et al., 2004; Sheng et al., 2011), classifying disease datasets (Huang and Zheng, 2006) and mining human gene expression modules (Engreitz et al., 2010).

The value of clustering methods has been established in various genetic studies, as reviewed by Jiang et al. (2004), as a means to group genetic variants according to their functional relatedness (D’haeseleer, 2005). In an example of using imaging as phenotypes, Sloan et al. (2010) applied a hierarchical clustering analysis on 834 SNPs and clinical and imaging phenotypes, including left, right hippocampal volume and gray matter density. The association between each SNP and each endpoint was first computed, and then the clustering was performed on the results, wherein both genotypes and phenotypes were grouped based on similarity. Subsequently, *p*-values for each cluster were estimated using bootstrap resampling. This study showed that (1) SNPs are frequently associated with imaging phenotypes and rarely associated with clinical scores and (2) most of the genes found within clusters are associated with either beta-amyloid production or apoptosis (Sloan et al., 2010). A noteworthy point of this study is that it combined a pathway-based approach and clustering analyses together, first by selecting SNPs based on pathways and then applying clustering on genotypes and phenotypes, and demonstrated that priori driven and data driven approaches can be integrated into one study.

## COMPONENT-BASED ANALYSES ON IMAGING DATA (CATEGORY 3)

Not only does the development of various neuroimaging techniques improve the precision of measurement of brain attributes, but it also stimulates the growth of analysis approaches. The most common imaging modalities include functional MRI (fMRI), measuring the dynamic brain activity based on blood-oxygenation-level dependent contrast; structural MRI, assessing the volume and density of gray matter, white matter, and cerebrospinal fluid; diffusion (tensor) imaging, depicting the white matter tract connections; and magnetic resonance spectroscopy, obtaining biochemical information about the tissues of brain. Furthermore, collecting multiple types of imaging data from the same individuals becomes a common practice in the hope of revealing additional information and increasing our knowledge. Thus, methods for multimodal analyses have also emerged and developed rapidly. Here, we limit ourselves to the component-based multivariate analysis approaches applied to imaging data, though there are many other multivariate approaches, such as unsupervised clustering, supervised pattern recognition, classification and projection, and others (Dimitriadou et al., 2004; Demirci et al., 2008; Hinrichs et al., 2009; Filipovych and Davatzikos, 2011).

ICA with various implementation algorithms (Cardoso, 1997; Hyvirinen and Oja, 1999; Bingham and Hyvarinen, 2000) and its modifications and extensions (Bach and Michael, 2002; Beckmann and Smith, 2004; Calhoun et al., 2005; Hong et al., 2005; Lin et al., 2010) are the most popular methods for multivariate analyses on imaging data. Several reviews have been offered to the imaging field (McKeown et al., 2003; Calhoun and Adali, 2006; Calhoun et al., 2009). Here, we briefly summarize the main points. A typical ICA model assumes that the source signals are not observable, statistically independent and non-Gaussian with an unknown but linear mixing process. Consider an observed *M*–dimensional random vector denoted by *X = [x*_1_, x_2_,...,x_M_]^T^, which is generated by the ICA model: *X = AS*, *S* is the source matrix. The goal of ICA is to estimate an unmixing matrix *W* such that *Y* given by *Y = WX* is a good approximation to the “true” sources. *Y* is called the component matrix. In the context of imaging data, components are the independent brain networks embedded in the observed voxels. Furthermore, when MRI data from multiple subjects, each with their own temporal dynamics, are of interest, several ICA based multi-subject analysis approaches have been proposed (Calhoun et al., 2001; Schmithorst and Holland, 2004; Beckmann and Smith, 2005; Esposito et al., 2005; Erhardt et al., 2011; Calhoun and Adali, 2012). We refer to recent studies by Calhoun and Adali (2012); (Calhoun et al., 2009) for a more detailed explanation. A recent addition is independent vector analysis (IVA), which is a generalization of ICA for analysis of multiple datasets (Kim et al., 2006). It takes a model of *X*^[m]^ = A^[m]^S^[^^m]^, Y^[m]^ = W^[m]^X^[m]^, where *M* is the number of datasets. Its cost function, the Kullback–Leibler divergence between two functions of dependence (joint probability density function of components and the product of marginal probability density function of components), allows maintaining the independency among components while increasing dependency of components between datasets (Lee et al., 2008a,b). Based on simulation (Lee et al., 2008b; Dea et al., 2011), IVA shows excellent performance in capturing inter-subject variability and the performance enhancement increases when the spatial variation of a given component across subjects is substantial.

For multimodal imaging analyses, a set of solutions with different emphases have been proposed and extensive reviews of these methods are also available (Biessmann et al., 2011; Sui et al., 2012a). Biessmann et al. (2011) reviewed the multimodal analyses from a variety of perspectives, including multimodal imaging study setup, the advances achieved in basic research and clinical applications, the methods for artifact removal, data-driven and model-driven analyses, and univariate and multivariate fusion. Sui et al. (2012a) focused on comparisons of the multivariate multimodal fusion methods rooted in ICA, canonical component analysis (CCA), and partial least squares (PLS) analysis. Similarity between methods fusing multimodal imaging data and multivariate analyses to bridge imaging and genetics are discussed in the next section.

## MULTIVARIATE ANALYSES BRIDGING IMAGING AND GENETICS (CATEGORY 4)

Given the characteristics of imaging and genetic data, multivariate multiple regression is a natural choice, where genetic variants are predictors along with other influencing factors such as age and gender, and imaging variables (regions or voxels of brain) are response variables. In practice with a set of SNPs and brain voxels (they are usually not independent to each other), regularization or modification of traditional multivariate multiple regression has to be taken in place. Wang et al. (2012a) proposed a group sparse regularization on multivariate regression. SNPs are grouped based on genes or LD blocks. A group sparsity to reduce to only genes or LD blocks relevant to all imaging phenotypes, and an individual sparsity to select only important SNPs are all enforced. Lin et al. (2012) presented a projection regression model that is also suitable for imaging genetics. The key of this model is to estimate the principal components of heritability (covariance between multiple phenotypes and genetics of interest), followed by a multivariate regression on the principle components.

When facing a very large number of genetic variants, such as genomic SNPs, and a large number of voxels in the brain, researchers in imaging genetics, very interestingly, has focused on a series of very closely related methods to capture interactive or integrated effects and possibly many genotype-phenotype pairs. These methods include PLS, CCA, reduced rank regression (RRR), and ICA (Hardoon et al., 2009; Liu et al., 2009b; Vounou et al., 2010, 2012; Le Floch et al., 2012; Meda et al., 2012; Chi et al., 2013). They are designed to simultaneously extract latent variables from both genetic and imaging data, which become new genotypes and phenotypes, and the connections of new geno-pheno variables are maximized using different cost functions.

**FIGURE 2:**
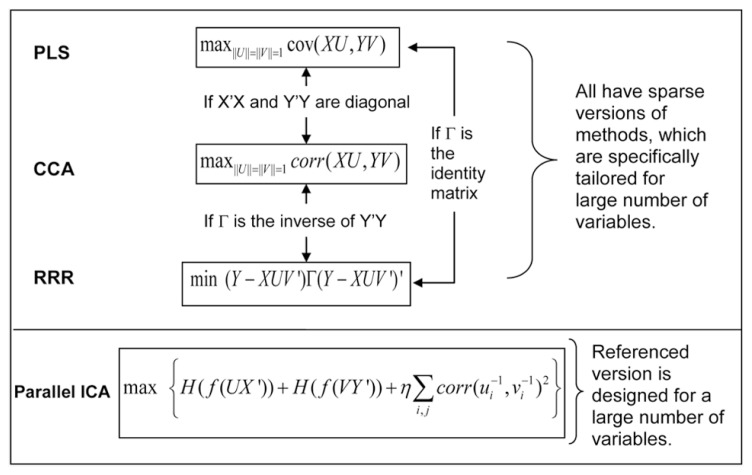
**Cost functions of four multivariate association methods, and their relation and extensions for a large number of variables**.

We can use a typical imaging genetic example to illustrate the relation of these methods. We denote by *X* an *n* × *p* matrix of genetic SNP data, and by *Y* an *n* × *q* matrix of imaging data, where *n* is the sample size, *p* is the size of SNP loci, *q* is the size of voxels, and *n << p* or *q*. The latent variables are obtained through projecting the* X* or *Y* to new directions formed by the vectors in *U* or *V* matrices. **Figure 2** plots the cost function of each method and the condition under which two different methods become equivalent. PLS maximizes the covariance between latent variables of the two modalities, while CCA maximizes the correlation between them. In a high-dimensional problem where the number of variables is significantly larger than the number of samples, it is common to assume that the covariance matrices of *X* and *Y* are diagonal (Vounou et al., 2010; Le Floch et al., 2012). Under such a condition, CCA and PLS become equivalent. The RRR model takes a more general formation that begins from a multivariate linear regression from *X* to *Y*, and reduces the rank of the project matrix, a product of *UV*′. Through minimizing the regression error noted as(Y-XUV’)Γ (Y-XUV’)’, RRR obtains the project matrices *U* and *V*. When the function of Γ is the identify matrix, RRR is equivalent to PLS, and when the function of Γ is the inverse of covariance matrix *Y′Y*, RRR is equivalent to CCA. Note that the core computations of PLS, CCA and RRR all involve single value decomposition so that the latent variables or projection vectors within one modality (genetic or imaging) are orthogonal to each other. In contrast, ICA emphasizes that latent variables (components) are maximally independent from each other, which can be optimized through many forms of statistical measures, including minimization of mutual information and maximization of non-Gaussianity. One extension of ICA methods applied to imaging genetics is parallel ICA, which simultaneously maximizes both the independence of components and correlations between projection vectors of the two modalities (Liu et al., 2008b).

Parallel ICA was first introduced into imaging genetics in 2009 (Liu et al., 2009b) when applied to a genetic study of schizophrenia with a 384 SNP array and auditory oddball fMRI data. Since then, this method has been made available for the public through the fusion ICA toolbox^8^. This approach has been utilized by various other groups (Jagannathan et al., 2010; Meda et al., 2010, 2012; Meier et al., 2012). A noteworthy point is that parallel ICA can also be applied onto other types of data in addition to genetics and images (Liu and Calhoun, 2007; Liu et al., 2009a; Wu et al., 2011; Meier et al., 2012). A simulation study showed that parallel ICA performs better within a certain range of sample size vs. genetic variable ratio (Liu et al., 2008a). When a genome-wide high-density large genetic array (e.g., >100K SNP loci) is in place with a relatively small sample size, new extensions of parallel ICA are proposed to improve the performance by incorporating prior information about genetic or imaging data called parallel ICA with reference (Liu et al., 2012a; Chen et al., 2013). As showed by Chen et al. (2013), this approach leverages prior knowledge of known genetic functions to guide ICA for specific components. Thus, a specific SNP factor centered at gene ANK3, which is a schizophrenia susceptibility gene (Ripke et al., 2011), was extracted from a large SNP array (>700K loci). While this method does help extract particular genetic components, which may not be extracted otherwise (Liu et al., 2012a; Chen et al., 2013), its performance relies on the accuracy of reference (Liu et al., 2012a).

As noted above, PLS, CCA, and RRR are closely related. They all introduced the sparse version of algorithms – sparse PLS (Le Floch et al., 2012), sparse CCA (Boutte and Liu, 2010; Chi et al., 2013), and sparse RRR (Vounou et al., 2010, 2012) – when applied onto a large number of variables in imaging genetics. Not only does the increase of sparsity make the interpretation more plausible, but also strengthens the stability of results by avoiding the over-fitting problem. Le Floch et al. (2012) showed through simulation that different levels of regularization on sparsity may produce different results for CCA and PLS, and the two methods converge together with the corresponding regularization strength. Similarly, for RRR, sparsity affects the performance (Vounou et al., 2010), and how to choose sparsity is critical in real applications. Up to now, only sparse PLS (combined with a filtering step) and sparse RRR have been applied to real imaging genetic data with larger than 100k loci (Le Floch et al., 2012; Vounou et al., 2012).

The differences among these methods besides mathematical models listed above also include settings in practice. First, the number of latent variables (components or ranks) to test is chosen differently. CCA, PLS, and RRR extract same numbers of components for genetic and imaging data, and pair-wise connections are tested. Though guidance is discussed for the choice of component number, users of these methods tend to be very conservative. Silver et al. (2012) only investigated the components from first rank in their RRR application, and Vounou et al. (2010, 2012) investigated the top three ranks. In the application of CCA, Hardoon et al. (2009) tested the top pairs of components, and Le Floch et al. (2012) examined the first two pairs of components for both CCA and PLS methods. In contrast, parallel ICA, following the principle of Infomax ICA (Bell and Sejnowski, 1995; Cardoso, 1999), first estimates the number of components embedded in genetic and imaging data. Estimation is either based on information theory (Akaike, 1974; Li et al., 2007) or stability (Chen et al., 2012a), with the goal of reliably, maximally explaining the variance of data. The number of components for genetic and imaging data can be different, and the pairs of related components between the two modalities are driven by data. Sometimes pair-wise correlations are not necessary (Meda et al., 2012). Judging from this aspect, parallel ICA carries advantage of exploring more possible connections between the two modalities, while other methods target only the top correlated components.

Second, all methods are limited in handling a large number of variables (particularly SNP loci). CCA, PLS, and RRR methods may run into over-fitting problems, where cross evaluation performance drops (Le Floch et al., 2012). Parallel ICA fails to identify the connections between modalities (Liu et al., 2008a). The ways to overcome this limitation are also different. Pre-filtering SNP loci to reduce the dimensionality is successfully implemented for CCA and PLS. Le Floch et al. (2012) presented a comprehensive comparison of PLS and CCA combined with different filtering methods. They showed that incorporating a filtering step before the multivariate association test (with the goal of removing irrelevant SNPs) can improve the performance for both methods. Their real data application makes clear that the dimension reduction (which reduced 700k SNPs down to 1000 SNPs) is an important step for avoiding over-fitting with such large genetic data. Although various means can be used to pre-filter SNPs, we recommend leveraging large population genetic data as a reference, such as Psychiatric Genomics Consortium^9^. For RRR, enhancing the sparsity to select only a small number of SNPs is an effective way to increase stability. Yet, the choice of sparsity is not easy (Vounou et al., 2010). N-fold cross evaluation can be used to decide the best parameter. Vounou et al. (2012) chose to test a range of sparsity settings and select resultant SNPs with high probability. Parallel ICA leverages prior information (a referential SNP set) to increase chances of extracting relevant genetic components associated with imaging phenotypes from large SNP data. The difficulty with this approach lies in how to decide the reference. In particular, what we should do when we do not have any prior knowledge about genetics regarding a particular phenotype? While prior information helps interpret the genetic result in a degree, parallel ICA need to threshold the resultant latent variable to select the most weighted SNPs, since no sparsity is in place (Chen et al., 2013).

Third, verification of results from latent variables is very important to guard against false discoveries. N-fold cross evaluation has been utilized for CCA and PLS, and sub-sampling is used in RRR, not exactly verification but increasing the stability (Silver et al., 2012; Vounou et al., 2012). Permutation and leave-one-out evaluation are used in parallel ICA (Liu et al., 2009b; Chen et al., 2012b). We strongly recommend future users to incorporate certain verification steps in their studies, given the complexity of the methods mentioned. To date, only parallel ICA has a ready-to-use package available^10^.

Except for multivariate analyses based on latent variables, methods in machine learning category, i.e., training algorithms with known knowledge and using them to predict the unseen data, have also been applied to imaging genetics. For instance, support vector machine on ICA factors of genetic and fMRI data together achieved better separation of schizophrenia patients from controls than using either type of data alone, suggesting that genetic and brain functions capture different, but partially complementary schizophrenic features (Yang et al., 2010). Within the same line, Wang et al. (2012b) proposed a multimodal multitask learning algorithm that combines genetic and multimodal imaging features to predict simultaneously diagnoses and cognitive function. In this algorithm, classification and regression are performed jointly, and a group L1-norm regularization is used for feature selection to integrate heterogeneous imaging genetic data. One of strengths of this approach is that genetic markers and imaging biomarkers relevant for both diagnosis and cognitive function are identified. Another new application of learning algorithms in imaging genetics is random forest on distance matrices, where by employing distance measures between input variables, various interactions (away from original space) are modeled and random forest search is used for selection of best sets of features (Sim et al., 2013). While it provides promising results, the requirement for intensive computation and sophisticated modeling may hinder further applications, which is true for other methods too.

## CHALLENGE AND FUTURE DEVELOPMENTS

During the last decade, imaging genetics has rapidly developed into a promising, high impact research field and extended into a body of studies on mental disorders, including both human and animal studies. As Meyer-Lindenberg (2012) stated, future imaging genetic studies have to confront the complexity of epistasis, pleiotropy and gene-by-environment interactions, and this issue will become even more pressing as the field moves into whole genome sequencing. Although methods reviewed here attempt to tackle this complex problem, limitations are clear. For example, none of the methods can really address the genome-wide whole brain association without filtering or dimension reduction. Some multivariate methods such as MDR and prior knowledge guided approaches have not been fully incorporated into imaging genetics yet. Methods of CCA, PLS and RRR, facing over-fitting issues when handling large genetic variables, may be improved by leveraging prior information. Methods of parallel ICA may need to enhance sparsity within the independent genetic components. Such limitation in fact relates to a common problem across multivariate analyses, which is the difficulty in interpreting results (i.e., results are lack of direct biological meaning). For instance, GSEA does not model the exact interaction among SNPs. The latent component does not necessarily hold direct biological reason why multiple genetic variants form into one factor, or why hundreds of voxels group into one brain network. One way to alleviate this problem is to incorporate additional information, such as known biological information, cellular level information, or behavioral specific information, into analyses. Further developing current methods and integrating more information will continue to be an important research frontier.

As matter of fact, another pressing demand raised by Meyer-Lindenberg (2012) in the future of imaging genetics is to integrate various types of data relevant to imaging genetics, beyond just two modalities. The new data can be proteomic, gene expression, epigenetic, behavioral and environmental variables. Studies have shown their relevance to brain structural and functional changes, genetic mutations, and psychiatric disorders (Clark et al., 2006; Serretti et al., 2007; Maric and Svrakic, 2012; Liu et al., 2013). The relationship among these data is by no means simple and pair-wise. To date, very few methods have been applied in imagine genetics to tackle the relation beyond two modalities (expect for *post hoc* analyses with behavior or diagnosis). It is very promising to see that some studies have stepped into this direction, though only for multimodal imaging data (Correa et al., 2010; Sui et al., 2012b). How to integrate such data in a systemic way with embedded biological hierarchy is still an untouched land. Methods and models incorporating multiple levels of biological variables (here including behavioral or environmental variables) into broader imaging genetics are another research direction of great potential and impact.

To date, very few studies focused on CNV’s effect on brain-based phenotypes (Yeo et al., 2011; Boutte et al., 2012; Liu et al., 2012b), even though many studies have identified a relationship between CNVs with psychiatric disorders (McCarroll and Altshuler, 2007; Bassett et al., 2008; Guilmatre et al., 2009). Meyer-Lindenberg (2012) has indicated that the future of imaging genetics will recognize the importance of the sizeable amount of variation in CNVs. Given the low incidence of individual CNVs, in particular large and rare CNVs, such studies are more likely from multi-site collaborations, where increasing numbers of imaging genetic studies are heading for (Schumann et al., 2010; Thompson et al., 2014). Methods to encompass data from multi-sites, controlling for not only different equipments or experiments but also different local populations or environments, are in great need, which have to consider both computational feasibility and mathematical (model) validity.

Given that the future focus of imaging genetics is expected to be multi-site, large scale, genome-wide whole brain, multiple level association studies, we believe that more effort should be focused on the development of methods that can confront these challenges.

## Conflict of Interest Statement

The authors declare that the research was conducted in the absence of any commercial or financial relationships that could be construed as a potential conflict of interest.

## Footnotes

1 http://fsl.fmrib.ox.ac.uk/fsl/fslwiki/

2 http://www.fil.ion.ucl.ac.uk/spm/

3 http://afni.nimh.nih.gov/afni/

4 https://surfer.nmr.mgh.harvard.edu/

5 http://pngu.mgh.harvard.edu/ purcell/plink/

6 http://www.openbioinformatics.org/penncnv/

7 http://www.broadinstitute.org/scientific-community/science/programs/medical-and-population-genetics/birdsuite/birdsuite

8 http://mialab.mrn.org/software/fit

9 https://pgc.unc.edu/

10 http://mialab.mrn.org/software/fit/
